# Understanding mental health through computers: An introduction to computational psychiatry

**DOI:** 10.3389/fpsyt.2023.1092471

**Published:** 2023-02-07

**Authors:** Juan Camilo Castro Martínez, Hernando Santamaría-García

**Affiliations:** ^1^Departamento de Psiquiatría y Salud Mental, Facultad de Medicina, Pontificia Universidad Javeriana, Bogotá, Colombia; ^2^Ph.D. Programa de Neurociencias, Departamento de Psiquiatría y Salud Mental, Pontificia Universidad Javeriana, Bogotá, Colombia; ^3^Centro de Memoria y Cognición Intellectus, Hospital Universitario San Ignacio, Bogotá, Colombia; ^4^Global Brain Health Institute, University of California, San Francisco – Trinity College Dublin, San Francisco, CA, United States

**Keywords:** computational psychiatry, computational phenotype, precision psychiatry, translational psychiatry, computational modeling

## Abstract

Computational psychiatry recently established itself as a new tool in the study of mental disorders and problems. Integration of different levels of analysis is creating computational phenotypes with clinical and research values, and constructing a way to arrive at precision psychiatry are part of this new branch. It conceptualizes the brain as a computational organ that receives from the environment parameters to respond to challenges through calculations and algorithms in continuous feedback and feedforward loops with a permanent degree of uncertainty. Through this conception, one can seize an understanding of the cerebral and mental processes in the form of theories or hypotheses based on data. Using these approximations, a better understanding of the disorder and its different determinant factors facilitates the diagnostics and treatment by having an individual, ecologic, and holistic approach. It is a tool that can be used to homologate and integrate multiple sources of information given by several theoretical models. In conclusion, it helps psychiatry achieve precision and reproducibility, which can help the mental health field achieve significant advancement. This article is a narrative review of the basis of the functioning of computational psychiatry with a critical analysis of its concepts.

## Introduction

The brain has been conceptualized as a computer performing continuous calculations about itself and its environment. Moreover, according to the theory of systems and Bayesian approaches, the brain is conceived as a complex, non-linear computational device (1, 2). The mentioned approaches could benefit a further comprehension of multiple levels of analyses that subsume mental health and psychiatric diseases.

In the psychiatry field, various attempts have been made to understand mental health and disease fundamentals. However, those intents have generated different explanations within multiple theoretical models, which are often disconnected and lack of complex understanding of mental health and psychopathology integrating many levels and systems. Thus, a point has been reached where a paradigm shift is needed. Dimensional and transdiagnostic levels of understanding are required to better comprehend. Some of the possible answers have chosen the use of mathematical principles to reach a multilevel analysis and generate hypotheses that can be validated. Such an approach provides the possibility of achieving a unifying theory, increasing accuracy, and reproducing what was found previously by other authors. In this context, computational psychiatry is a tool for precisely this purpose. It should be clarified that this probabilistic view of the brain is open to controversies (3).

Psychiatry has always encountered multiple controversies during its history. These, in turn, have generated multiple internal and external crises that have questioned its validity as a science and its management of mental illness (4–6). These criticisms have focused primarily on the validity of their concepts and constructs (7), their diagnostic capacity (8), the reliability between different observers, and the lack of biomarkers to determine the diagnoses, treatments, and prognosis of the condition (9, 10). Additionally, they have focused on the variability of the course of different disorders, typically heterogeneous in their presentation (11).

Psychiatry has used various approaches to overcome these criticisms, such as nosological formulations. This strategy attempted to elucidate their biological basis (12) by achieving greater reliability in the diagnosis. Such systems have generated multiple syndromes with significant heterogeneity in their course, clinical manifestations, prognosis, and response to treatment but grouped under the same diagnostic category (13). This has raised the possibility that they also have a different neurobiological basis. It has also shown the limits of these tools. However, a myriad of empirical data has been obtained through such systems. Although, this data suffer from poor integration of cellular, synaptic, neuronal circuitry, and complex behavioral responses (14, 15). For example, there are strong interactions between genes and environment at the genetic level, but no clear paths to how these develop into a specific phenotype. This also occurs in neuroimaging, where only indirect measurements of the behavioral variables observed in clinical practice have been achieved (15). In conclusion, a cohesive model capable of taking data from different sources and giving adequate weight to each source of information has yet to be reached.

Computational modeling of behavior was elaborated by specializations of the neurosciences, which preceded computational psychiatry. One of the first to do it was computational neuroscience. It is responsible for studying the brain at a theoretical level, determining the principles and mechanisms that guide the development, organization, and process of information (16). This is achieved using computational models (descriptions and explanations of processes) that occur at different spatial and time scales and with non-linear interactions. More specifically, computational neuroscience makes hypotheses about the processes that operate in the brain at different analysis levels and unites them to corroborate them. The goal is to understand the functionality of complex systems such as the brain, formulating quantitative hypotheses (17). In this context, computational models give a practical tool to address specific brain characteristics, such as its emerging functions. Depending on the question type one wants to answer; one will opt for a particular abstraction level to form a model. Here, the three levels of analysis proposed by Marr and Poggio are relevant (18). These are the computational level (the “why”), which is the most abstract and deals with logical-mathematical reasoning; the algorithmic level (the “what”), which evaluates the rules of the process; and, finally, the level of implementation (the “how”) (19). For this author, brain research was conceived as a problem of information processing (17).

Based on this, computational psychiatry has appeared as a way to achieve this integration. Computational psychiatry uses formal models of brain function to characterize the mechanisms of different psychopathological manifestations by describing them in computational or mathematical terms (20). This facilitates the study and articulation of these data by incorporating knowledge from other sciences such as cognitive science, computational neurosciences, and “machine learning” (20–24), trying to translate knowledge between different levels of analysis. This review aims to give a comprehensive view of the foundations of computational psychiatry, highlighting its interactions with different approaches like biophysics and evolutionary psychiatry to arrive at precision psychiatry.

This field has become an essential tool for finding novel solutions, encompassing both the context and the individual. In addition to providing investigative and practical means to arrive at response to specific needs in these contexts in a cost-effective way. Nevertheless, it is necessary to understand its foundations and how it applies to research and clinical purposes.

In this scoping review, we first describe the importance of computation modeling in psychiatry to face limitations from a system theory perspective. Then, we explain how computational models are built, giving particular emphasis on their underlying concepts. Moreover, we comprehensively explain the statistics surrounding the computational models and their applications at different levels of plausible explanations in psychiatric scenarios. Finally, we reflect on model validation and the potential limitations of computational psychiatry.

## Methods

A narrative review of the literature was conducted, focusing on computational psychiatry’s fundamental concepts and applications. To this end, we searched PubMed, MEDLINE, EMBASE, and EBSCOHost for both narrative and systematic reviews of computational psychiatry using the terms “computational psychiatry,” “biophysical psychiatry,” “computational modeling,” “digital phenotyping,” “precision psychiatry,” and “computational neuroscience.” After this search, essential studies were also reviewed within the articles’ references. Articles written in English and Spanish were selected. Articles based on their publication date were not excluded. The last search was conducted on 30 December 2022.

## Need for comprehensive models of mental illness

A model is a heuristic way of understanding complex interactions and their relationships by employing a simple rule (25). In the case of mental health, modeling used have degenerated into a diversity of disjointed data from various theories. Additionally, modeling through the relation between brain activity and psychiatric phenotypes has many pitfalls because they are focused and predict complex profiles rather than unitary cognitive processes (7, 25).

Most explanatory models used in psychiatry and psychology focus on narrative methods, with the problem of approaching human behavior from only external behaviors or epiphenomena conducts without ever finding a clear biological causal or mechanistic basis (7, 25). This approach leads to difficulties in determining clear biological and clinical processes (25) with implicit categorical errors (26). One example is the measures based on self-reports with poorly defined variables and poorly elucidated pathophysiological mechanisms (14). On the other hand, by not knowing the mechanistic or robust theoretical approaches to study mental disorders, some studies are initiated to look for relationships with multiple variables in so-called “fishing expeditions.” In turn, this can generate associations that do not reflect the actual phenomenon (27).

Moreover, theoretical models have often been chosen for data-driven approaches. However, this theoretical approach can exhibit challenges when studying human behavior as they assume *a priori* hierarchies assessing predictors of an outcome and can be restricted to a partial understanding of a complex model (25). Finally, one can obtain data replicated by others, assuming a certain degree of validity, which could ultimately be wrong. In fact, replicability issues represent one of the biggest current challenges in psychological studies (25).

## Computational models applied in neuroscience and psychiatry

Neuroscience and psychiatry lack methods for constructing, assessing, and validating theoretical models, which should be more extensive than describing relationships between different variables (25). Against this issue, computational modeling of dynamic systems becomes vital as it allows the generation of data-driven validations of conceptual reference frameworks and biological measures, avoiding issues due to spurious statistical associations and biases in building models (25, 28).

Thus, computational models help simultaneously manage massive information sources and articulate biological, psychological, and contextual models for understanding human behavior. Different approaches as machine learning, deep learning, and explanatory modeling (13, 29), help to process information building in models only determined by data avoiding theoretical and restriction biases.

An important method computational psychiatry uses is differential equations, which express neurobiological systems’ functioning more closely. They represent changes as a function of time codified by the interactions with other non-linear variables (25). Consequently, they can join several equations that mathematically specify relationships between symptoms, environmental factors, and neurobiological substrates (28, 30, 31). This exemplifies the possibility of reconciling different perspectives and empirical data, providing cohesive, stratified models for understanding complex phenomena.

A big group of mental disorders computational models focuses on altered learning and decision-making processes as the central components (15), highlighting the relevance of information integration processing. These learning models have been used in computational cognitive neuroscience, using tools like machine learning to model a specific phenomenon. These cases are usually divided into supervised, reinforcing, and unsupervised. Within these models, it is assumed that the objective of learning is to form storages of representations to be remembered and guide behavior, although the mechanisms to perform it may differ according to the model (29). In the case of supervised learning, specific feedback is received after the experience.

In contrast, in reinforcement, this feedback is not explicit and can be delayed and influenced by multiple factors. In addition, it can be done in the form of punishments or reinforcements, which may not be directly associated with the behavior. Finally, in unsupervised learning, the subject is the one who must make sense of the experience without any feedback.

Another use of the computational approach in psychiatry is modeling a specific phenomenon. To achieve this, computational models offer a tool to facilitate it *via* the generation of self-generated models that synthesize data through the sampling of inputs and achieve an approximation of specific outcomes, thus integrating Bayesian probability (15, 32–34). Ultimately, they enable us to make a probability distribution and hierarchization of the best predictors of a neural, cognitive, or behavioral state among massive interactions from different sources of variables (27). In psychiatry, mentioned models would help us elucidate and better understand psychopathological phenomena by relating them to neuronal processes and their normal function (35).

This step can be performed at different levels of explanation, such as at the molecular level, neural networks, cognitive processes, or mental symptoms. Computational models now allow us to predict symptoms and clinical presentation of neuropsychiatric disorders by studying brain volume information and functional connectivity networks *via* data-driven methods (machine learning procedures, support vector machine methods, or deep learning approaches) (32–34). Moreover, these approaches have been relevant for biophysical psychiatry (14), where psychiatric phenomena of interest, such as psychopathology, relate to alterations in the biophysical properties of, for example, the membranes of neurons, as seen in other reviews (14).

Moreover, computational methods have helped to understand the impact of different sources of information in neurodegenerative disorders (36) or elucidating individual and contextual factors determining complex behaviors such as violence (37).

## Statistical foundations

Computational theories of the mind are based on probabilistic perspectives. The brain processes are considered mimicking computational functions of the system to infer the state of its environment and decide which course of action to follow (35, 38). The inputs will never be completely reliable, so there will always be uncertainty that has to be considered when performing any task. Therefore, Bayes’ theorem (the combination of the initial expectation of the state of the environment and the probability of the input determining a modified estimate of the state of the environment) is used to describe these processes (15, 35). Describing in such a way brain processes can be translated to computational psychiatry approach. This contrasts with the statistical approach used in psychiatry, which asks about the probability that the data have resulted from the null hypothesis (25) and corresponds more to discriminative models (35). By contrast, in the computational psychiatry perspective it is possible to assess different layers of biological, psychological, and social-contextual information and use algorithmic approaches to assess multiple interactions between layers, modeling data and testing those models with complex validation processes of findings.

Different interactions can be found when computational approaches are studied in neuroscience and psychiatry. First, some computational models in neuroscience accept the metaphor of the brain as a computer (20, 39). Mainly, the models who accept the metaphor of the brain as a computer describe brain biological processes as part of a computer that primarily formulates predictions on future states based on massive integration of past interoceptive and exteroceptive information. In this perspective, the brain is an entity that constantly builds and updates a model of reality through sensory inputs named generative (40). The optimization of this generative model must lead to the minimization of free energy (the energy used by the brain) (2). Moreover, the brain tends to formulate different predictions and minimize errors to curtail energy expenditure, which can be done in two ways: either by adjusting the cognitive scheme of the world or by changing the pattern of action (15). The latter is essential since it can explain psychopathology, including functional neurological symptoms (41).

Computational psychiatry is aligned with previously mentioned notions, as it integrates different levels of information to formulate appropriate models to describe and understand mechanistically healthy and pathological behaviors. Moreover, computational psychiatry performs predictions of potential states and biomarkers and runs test and retest validations assuming complex heuristics to predict psychiatric phenotypes (18, 35, 42). The goal is to generate accurate and robust predictions with the minimization of the workload to reach meaningful outcomes.

According to Breiman (43), two statistical models are used in the mentioned approaches. The first is algorithmic or “data-driven” models, aiming to predict results by having a specific group of data (inputs) following complex statistical procedures leading to massive interactions between variables. The second model is described as “theory-based” modeling, where a pattern of outputs and initial data is used to determine how the process is performed to generate this data (35, 44).

Both types of statistical analysis proposed by Breiman share statistical tools and can be associated with concepts from the learning field through reinforcements. Thus, it offers different visualizations as to how to conceptualize the computations or calculations made by the brain (15). Also, it can give way to the use of methods like machine learning in computational psychiatry (21). Learning is a complex process since there is always uncertainty. A specific behavior is selected according to the reinforcers and punishments values during reinforcement learning to maximize a particular outcome. All this is based on the prediction error measured utilizing the learning rate. The impact of this error depends on its accuracy (inverse uncertainty) (45).

There are two different ways in which experience is used to estimate and predict future rewards and punishments. The first is a model-based cognition, also called goal-directed, where experience is compiled into a generative world model. This involves the inference of future possibilities, generating an enormous computational cost. This contrasts with model-free cognition, where no information about the change suffered is stored but only encodes how much reinforcement is obtained when the subject is in a state or performs a specific action. In the latter, computational costs are decreased, but at the cost that the system becomes slow and inflexible, with no possibility of responding to changes in the environment (46).

To abridge previous gaps, the predictive coding model is important, where a unit at a specific hierarchical level sends messages to one or more units of lower levels that predict its activity (47, 48). The discrepancies generated between these predictions and the actual input are then passed to higher levels of the hierarchy as prediction errors. They are then reviewed to refine the prediction (35). The uncertainty (inverse precision) of each level determines the rate of learning at each level, determining the size of adjustments that must be made to explain the data that has been sensed. This approach is closer to the representation of the nervous system, a dynamic and hierarchical system. However, this hierarchical model has come into question with models such as the heterarchical model (49, 50), where the components of a system do not have a specific order. However, they can have different connections depending on the function and the context that is being analyzed.

Statistical models based on data could give important tools for clinical practice. One is SpeechGraph, a computational tool that can quantitatively assess a patient’s discourse structure through graph theory (51, 52). This tool does not take the process of speech formation (the syntax). However, it is possible to calculate the attributes of the graph created from the discourse and, through these, can differentiate a control from an affective and non-affective psychosis (52–54). It can also determine the differences in the development of the discourse longitudinally of children with psychosis and controls (55) and in cases of dementia, these can be correlated with other cognitive deficits (56). The importance of these approaches has been taking force with Natural Language Processing (NLP) associated with machine learning paradigms (57, 58). These models can evaluate specific parts of a complex neurocognitive process like language and then aid in comprehending the underlying pathological mechanisms.

In the statistical models used in theory-based models, the parameters are surrogate variables of neural computations (processes). In this case, the parameters do adapt to neurological or behavioral data. Therefore, this model can be used to elucidate possible dysfunctions underlying multiple mental disorders (59), such as the search for pathophysiological processes underlying transdiagnostic alterations. This theory-based approach can also account for neurocognitive approaches like the Bayesian active inference model of discourse. The person speaks, and this person monitors internal and external signals in the search for errors (60) and explains the way social cognition alterations could disrupt language emission or reception.

To this end, several conditions must first be secured. The first thing is that the model must be able to predict multiple experimental data. To determine this, the effects of the parameters on the model’s predictions must be independent, and there must be sufficient data. These conditions are mainly used to compare different models to determine which fits best to a particular phenomenon. One of the ways to do this is to simulate data in each model one has and determine the ability of each model to generate the “real” data of the variable being studied. Remember that the empirical and predicted data will not coincide perfectly (59). Then, these parameters may be used as computational markers of psychiatric illnesses. These markers associate psychiatric dysfunctions with failures in neuronal computations (predictive coding, divisive normalization, and contextual modulation). With these markers, what one is trying to do is (59): (i) Distinguish between diagnoses with similar symptom profiles: spectrum problem or symptom overlap. (ii) To characterize heterogeneity within diagnostic categories concerning alterations in computational mechanisms. (iii) Predict relapses or responses to treatments.

Although brain processes result in great complexity, they present a hierarchical organization that allows them to be broken down into more basic operations and easily understood. In the same way, phenomena studied by psychiatrists can be simplified and organized in hierarchical models through factor analysis (61) or network theory (62).

## Levels of analysis

Overall, this data processing method seeks to integrate neurological, psychological, and social reference frameworks. Indeed, it searches for a way of making bridges between different levels of this hierarchical organization of the brain, which has to consider its surroundings as described by the concept of the *phantastic organ* (21).

It is then possible to make models that describe the molecular basis of the individual neuron, describing its electrical properties and the generation of the action potential. This is done by employing a set of equations that describe its properties (14). To achieve this level of characterization, previous studies of significant impact on this understanding are taken as the description of the signal propagation by neurites (63) and others (64).

Also, within the first level of analysis, one can opt for the genomic analysis and description of the studied phenomena. Genomic approaches attempt to determine the biological relevance of genetic variants and predict their influence on the phenotype (65). This review shows that computational models lend themselves precisely to validating and confirming biological relevance. Currently, the discovery of possible risk variants using GWAS (66–71) is much faster than their validation. Nonetheless, computational approaches have been developed for the prioritization of disease-gene candidates (72). This advancement has enabled researchers to elucidate co-expression patterns through network analysis (73).

Before continuing, some clarifications must be made regarding the conceptions of circuits at the neurosciences and the clinical level. The term circuit in neuroscience refers to microcircuits where biophysical processes modulate a response. Meanwhile, in clinical neurosciences, these are dynamic systems defined by control systems (25). This latter definition describes better the networks which are studied in psychiatry.

However, to discover circuits associated with a specific phenomenon, a hybrid approach must be used where the discovery of a circuit is based on observing the dynamics of its outcomes. This is then put into a differential equation that describes the system’s mechanistic structure. With this, a differential equation that describes the system’s response can be generated starting from the inputs and outputs. This is called the “transfer function.” At this time, machine learning can be used to discover plausible or related biological circuits. These circuits can be added according to their interactions generating complex systems (25). This kind of approach enabled researchers to develop theories of the function and associations of specific brain regions like the hippocampus (74) and, with this information, able to put forward hypotheses of different pathologies (75).

At the level of circuits, an attempt is made to elucidate the intrinsic neural activity evoked through different brain systems. These models incorporate the properties of neurons and synaptic connectivity. However, they are limited by the strategies used to acquire information from these networks, based on imaging studies. So, these models describe the brain as a network of interconnected nodes (76). To achieve this, there is a necessity to describe the structural connectivity matrix together with an equation that determines the neural dynamics of each node. Direct connections and the background activity of the area will influence these. Many of these aspects require biophysical knowledge at the molecular and cellular levels to achieve a more accurate approach to empirical neural dynamics. Furthermore, they could integrate with data and knowledge taken from the connectomics fields. In doing so, these approaches are helpful in investigating alterations in the brain connections in specific diseases (77) or arrive at transdiagnostic alterations (78).

As for the psychopathological level, examples are scarce. However, computational psychiatry can also be used to reach its understanding and even form practical applications based on psychopathological alterations of the computational level of information processing (79), such as salience processes. This opens the possibility of evaluating neurocognitive domains to evaluate a patient, which is currently underused for patients with psychiatric ailments. Within this part of the diagnostic and therapeutic process in psychiatry, various problems previously highlighted in terms of the validity of the psychopathological evaluation and nosological classification can be tackled (4–6, 80–84). Within this panorama, psychopathology can be considered a complex system (85), where alterations in its balance generate a search for homeostasis through an orientation toward the environment and a manipulation of its parts, reaching emerging qualities, which can be expressed as symptoms during a mental examination. Because of this, computational processes are privileged to achieve new perspectives that allow the clinician or researcher to overcome these obstacles.

Moreover, there is a possibility of considering constructs that may not be psychopathological but do contribute to suffering, such as domestic or gender violence. Nonetheless, different models have been proposed to tackle this problem, like the Hierarchical Taxonomy of Psychopathology (HiTOP) (86) and the network theory (81). This is how different diagnostic approaches using computational methods have been proposed (71).

However, to achieve this, different levels of analysis contribute differently to a specific phenomenon. Nonetheless, their integration is difficult to achieve, as well as the identification of a level more essential to the phenomenon studied. So, depending on the question to be answered, specific methods must be used to address it. Consequently, certain analysis levels will also be used preferentially. The problem lies in recognizing which level permits having a bigger and better picture of the studied event, weighing each component differentially. In other words, according to the question to investigate, certain elements of the phenomenon will be more important (essential) than others (87). In such a way, certain levels of analysis will carry more information within this question.

Finally, this must also be complemented with a longitudinal perspective (88–91), in which importance is given to how these processes will shape neurodevelopment (92, 93), where both normal and abnormal trajectories of such development must be studied for the possible determination of useful biomarkers or the understanding of the interactions that are at play and that can be associated with both normal and abnormal development. All this is associated with perspectives promoted by the RDoC initiative.

Considering the above, there are three types of perspectives to approach the description of dynamic systems, such as mental processes (25):

1. “Bottom-up” biophysical approaches: begin in individual neuronal functioning and are extrapolated to other levels of hierarchical organization, such as networks. In this case, the equations represent the properties of neurons, synapses, or ion channels. These observations can then be transferred to the functioning of neural circuits. However, the subsequent step between circuits and behaviors is much more complicated to surpass. There is an underlying problem: the whole can be greater than the sum of its parts. So, understanding the basic processes does not always arrive at a corresponding process in the higher levels of the hierarchy.
2. “Top-down” approaches, where one starts with an emerging phenomenon and tries to infer the set of neural mechanisms on which they are based. In this part, connectionist models used in cognitive and psychiatric neuroscience become essential. These models study neural systems that are involved in various cognitive processes. They attempt to arrive at the functioning of neural networks on a large scale and thus be able to achieve behavioral predictions (12). Models incorporating more than neurobiological systems are included in this part of modeling, such as social interactions or cultural influences.
3. Theoretical-informative approaches, where structural strategies are investigated where the brain can optimize the efficiency of information propagation based on graphs or network theory considerations.

Now, this complements modeling levels suggested by computational neuroscience (35), described in the introduction.

## Model’s validity

Validation requires integration of multiple sets of data involving different biological, psychological, social, and contextual levels of analysis. Computational modeling in psychiatry maximizes the amount of information predicted using data-driven hypotheses and testing processes *a priori* assumptions using a small pool of data (25). Another advantage of computational psychiatry is the capacity to generate, test, and validate available models or those generated in the research process (25).

Modeling validation can be tested using statistical parameters, including accuracy, sensitivity, specificity, and power measurements. The accuracy of a model is critical to take into account as it allows a certain degree of confidence in the results obtained. This is determined by the degree of error between the prediction it makes and the empirical data obtained. Meanwhile, the model’s power is evaluated by the diversity of inputs (different perspectives) and the time during which the predictions are valid (25).

Thinking of the brain as a machine that solves inferential problems can be an excellent way to generate testable computational hypotheses about psychiatric disorders (35) or even mental issues. Moreover, this is especially important because each measurement can have multiple explanations (multi-causality). The problem lies in finding which description is the one that best fits the data taken and enables a better prediction of future problems. For this step, the researcher can determine the model parameters that maximize the likelihood of the data given the model in a process known as model fitting. This likelihood is then used to calculate a quality-of-fit criterion (94). There will also be a degree of uncertainty, and there will always be room to improve the models. Then, a balance must be made between the complexity of the model and the model’s accuracy. A highly complex model leads to greater difficulty in achieving the understanding one wants to have of the phenomenon studied (95). But mental processes are highly complex, and some complexity of the model is inescapable. Alternatively, simple models can lead to poor prediction, in other words, lower accuracy and thus low usefulness. In addition to this, it must be considered that psychiatric disorders are characterized by their heterogeneity, so there may be several mechanisms at play in the same patient despite having the same phenomenological or nosological representation, which must be considered within the validation process (15).

As this computational modeling field grows, there is also the need to be able to compare different models. One such way to do so is using Occam’s law (94). Similarly, as the free energy principle governs the brain, one could select a model according to its predictive performance (its ability to predict observed data). Nonetheless, this approach is not enough for selecting theories. In this scenario, the model’s generative performance becomes a better way of selecting the model by falsifying it (94). The latter requires the simulation of candidate models in a denominated model recovery process. These two selection models are complementary an allow researchers to reach the most accurate modeling to explain a dataset (94).

Finally, the greater complexity and computing power put forward another issue: reproducibility. For an article to be reproducible needs that researchers share its data and coding, and in executing the code with the data given, one arrives at the same results. The ability to analyze more complex interactions between non-linear factors and their dynamic interplay could bring the researcher closer to data with a low signal-to-noise ratio, with a possibility of identifying false associations (96). It is essential to point out that this is not unique to computational approaches. However, it is partly facilitated by multidimensional datasets which go through rapid, flexible, and automated analysis (97, 98), as in Big data approaches. To tackle this problem, sophisticated analyses are required. However, there is a lack of infrastructure and knowledge to support this task.

Nonetheless, initiatives have taken place to tackle these limitations, and various articles have been written to describe steps to take to achieve the goal of more reproducible research, like improving methodological knowledge and independent methodological support with the encouragement of collaboration initiatives and open science (97) and to develop a way of accountability (99). It is also important to bear in mind the bias-variance trade-off (100). There is a conflict between bias error and variance error which must be minimized while constructing a computational model. A bias error generates when the model is not capturing relevant associations, while a variance error occurs when the model is overfitting.

## Precision psychiatry

When talking about precision psychiatry, we seek to achieve a computational phenotype. This means achieving a model that best suits the empirical data of the subject or phenomenon. This allows for generating inferences at the individual level about the underlying computational mechanisms that govern what is observed in the patient, thus overcoming the opposition between the dimensional and categorical perspectives (12). This is of utmost importance in ethnopsychiatry since it allows to the generation of specific modeling of behavioral alterations, which can be outside the nosological categories. Equally important, they acknowledge the impact of specific environments in a person’s life. In doing so, a better understanding of the person and their contextis reached; and one is capable of offering the best possible therapeutic approach (individualized and person-centered).

However, the traditional form of research in psychiatry has allowed predictions of the average functioning and mechanisms of pathophysiology to be achieved in a defined group of patients, such as that presented in nosological systems. Nevertheless, the problem of proposing differential diagnoses arises. When a differential or comorbid diagnosis is suggested, the clinician must determine from the findings in the patient what is the specific pathophysiological mechanism or of more significant predominance in the individual (15), which is currently impossible. This would determine the best therapeutic intervention for the patient and their prognosis (101). This possibility of differentiation between diagnoses and spectra within the same diagnosis has been made possible through “generative embedding” (102), although only in research.

Another problem is to consider phenomena outside nosological systems, which also have a high impact on society. A clear example in the Colombian case is that of violence, from which multiple phenomena and complex social processes have been generated that have contributed to the mental health of a population (103). Still, they should be given more importance in the research on mental health, especially from the medical perspective (104).

## Clinical applications

Transferring all the previously described concepts to the clinical and practical field has been costly and time-consuming (105). It is one of the most critical efforts to test the usefulness of these approaches (87). This has multiple reasons, which could be summarized as that mental health depends on normal brain function and how it is related to modification and is influenced by the individual’s context. It is a form of circular causality. These models or tools must describe dynamic, hierarchical, and non-linear systems. This means that it is challenging to have a clear and concise understanding and comprehend these phenomena or disorders. However, approaches are trying to address this problem by creating a bridge between neuroscience and computational psychiatry with cognitive neuroscience. It is essential to highlight that computational psychiatry can be a valuable tool in searching for these basic computations and how they modulate and emerge innovative functions from an evolutionary perspective (38).

Currently, psychiatry is primarily based on nosology contingent on classification systems such as the DSM or the ICD. However, this approach can be complemented by a dimensional vision, where they are added to the psychopathological manifestations and dimensions given a value within a continuum in models like HiTOP (86). However, this value can be non-linear or interact or correlate with other dimensions by modifying the syndrome and making it extremely difficult to quantify the weight of a specific factor.

A clear example is the determination of suicide risk (106, 107). The risk factors are determined through previous studies, but the quantification of these is carried out at the level of the clinician’s judgment, and the scales have poor operational characteristics (106–108). In addition, all this is done from population data without considering the differential influence of these factors on the individual. Machine learning has been used to predict suicide attempts and deaths from clinical records (102). For this reason, a way is required in to integrate dimensional and categorial visions, which often escapes the possibility of the clinician within their daily practice (44). The difficulties in diagnosing, prognosis, and treatment of this type of patient are highlighted. To have a complete picture of these applications, the reader can refer to the review made by Huys et al. (44).

These first approaches are still only applicable to research, but they give glimpses of the utilities of this tool. On the other hand, one can have clear examples where the first steps have already been taken to achieve a translation of this knowledge. Some of these examples are available below.

### Data-driven approaches

1. *Diagnostic classification:* In this aspect, elements of “machine learning” can be used. With this, neuroimaging data can be analyzed by distinguishing clusters of specific symptoms with specific neurobiological substrates, as seen by Costafreda et al. (109) or Mota et al. (54, 110). However, problems such as determining comorbidity as completely different disorders continue without the possibility that they have defined diagnostic limits (111). Because of this, the usefulness of these tools requires testing their properties in ambiguous cases, where there are more significant difficulties in differentiating.
2. *Prediction of clinical status*: This type of application focuses on identifying markers to determine the stage where a particular patient is to describe prognostic or treatment features. This has been used in early psychosis to predict social outcomes in a high-clinical risk sample (112). In other examples, NLP can be applied to clinical records like psychotherapy notes to enhance prediction models for different clinically relevant outcomes like suicide risk (113).
3. *Prediction of treatment response:* This aspect corresponds to the need to improve the prognosis and the ability to identify the best therapeutic alternative with an individualized approach. In the specific case of depressive disorder, where it is evident that only two-thirds of patients have a response after multiple pharmacological attempts (114–116), identifying the characteristics that could collaborate in the treatment choice is required. It may be that the cases referred to as resistant are not but require differential therapeutic responses. However, it has been attempted to achieve different ways to characterize and predict treatment responses, such as quantitative electroencephalogram markers (qEEG) (117, 118) which were validated by other studies (119). In addition, methods based on neuroimaging results have also been used (120), which be associated with computational approaches for pattern classification. All these approaches have been shown in their early experiences to improve responsiveness.
4. *Choice of treatment:* As mentioned in previous section, not all patients respond in the same way to treatments, even if they are first line. But as made explicit above, there are no variables or individual characteristics of the patient to determine it, even though multiple pharmacogenetic studies have been done in some specific situations. At this point, numerous binary classifications can be used simultaneously to achieve this task. However, to be feasible, a specific group of paraclinical must be used (121, 122). It can be used, for example, in electroconvulsive therapy, where simulations of electric fields can be integrated with the current knowledge of neurocircuitry to individualized electrode configurations (123, 124) and in the Deep Brain Stimulation (DBS) field (125).
5. *Clustering of clinically relevant data:* In this approach, unsupervised methods are used to cluster together characteristics of the sample giving rise to dimensional factors that can inform the patient’s clinical status. An advantage of this approach is that it facilitates the interpretability of the results (100). This approach has been used to identify brain fingerprints in different disorders from neuroimaging data (126, 127).

In these different applications, the researcher can take various sources of information to give a more accurate picture of the patient (128). This complementarity exemplifies the possibility of the constructing of mechanism-driven knowledge from data-driven approaches (100). These applications could then be articulated with network theory to understand mental disorders revised elsewhere (62, 129).

### Theory-driven approaches

These are initially “fed” by multiple data found at various levels of research, exploring the relationships between them. At the level of psychopathology based on Bayesian theory, the psychopathologic symptoms can be structured in three different ways: solving an inappropriate problem correctly, solving a suitable problem incorrectly, or solving a relevant problem correctly but in the wrong context (130). Moreover, from this conception, an analysis and a possible union of knowledge of brain structure and functioning can be generated together with behavioral variables seen in clinical practice.

1. *The course of the disorder:* In this section, Goldbeter’s article can be an example (131). The author gives a model of mutual inhibition between two processes (depression and mania) to explain the cyclicity seen in the disorder. In this example, the model does not contemplate neurobiological processes at neurocircuits, synaptic, neuronal, or biophysical levels. Still, it achieves a conceptualization of a phenomenon of extreme importance, such as the cyclicity in bipolar disorder.
2. *Predicting risk of recurrence:* There are other examples where the researcher could take a specific marker like effort and reward tasks to determine the clinical status of a specific disorder. And later, decide on the treatment according to the information this marker gives the practitioner, like the risk of recurrence (132).
3. *Neurocognitive functions:* The models can be used to describe the function of neurocognitive functions and domains, like working memory (133). And it enables researchers to put forward theories and models of pathological alterations of these processes (75). These models can also be used directly in conceptualizing a disorder like obsessive-compulsive disorder (OCD) and linking it to neurodevelopmental processes (134).
4. *Pathophysiological processes*: This type of model can give insightful perspectives that integrate different levels of analysis giving rise to a comprehensive and integrative knowledge of the disease processes. There are multiple examples across multiple disorders like schizophrenia (135–137). This, in turn, could give information about possible therapeutic targets. Researchers could also create models for explaining and understanding mechanisms associated with the therapeutic response, like neuromodulation strategies such as ECT (138).

In addition, an integration of these two approaches can also be achieved. This is because theoretical models must be fed from previously collected data to construct a good model. But also, a mechanistic model can generate available data for constructing pragmatic tools that can be used in clinical practice. To show how this applies to a specific pathology (schizophrenia), refer to the article by Valton et al. (139). In Table 1, there is a list of the examples used throughout this review with a description of the approach used and implications and contributions for the field.

**TABLE 1:** Clinical applications of computational approaches.

Title	References	Study aim	Data analyzed	Computational approach	Conclusions/Implications
First symptoms and neurocognitive correlates of behavioral variant frontotemporal dementia.	Santamaría-García et al. (32)	Analyze neurocognitive correlates of patients with bvFTD who debuted with apathy or disinhibition.	Data from a group of patients and controls involving neuropsychological, clinical, and neuroanatomical data.	Data-driven approach using machine learning associated with a multivariate analyzes.	This study gives an example of the possibility of integrating different levels of analysis of data with a longitudinal perspective. The latter is achieved by the longitudinal approach to the study and the description of correlations of first symptoms and their evolution. This study assessed multiple levels of analyses by implementing support vector machine approaches.
Robust automated computational approach for classifying frontotemporal neurodegeneration: multimodal/multicenter neuroimaging.	Donnelly-Kehoe et al. (33)	Determine if by using atrophy and resting-state functional connectivity one could differentiate between patients with bvFTD and controls.	Datasets from participants in different regions of the world.	Automatic, cross-center, multimodal data-driven computational approach using machine learning.	The multimodal approach explored in this study enhances the system’s performance in a multicenter protocol. This underscores the possibility of clinical applications in real-world conditions. This study implemented different machine learning models to abridge different levels of neurocognitive and clinical information in dementia.
At the heart of neurological dimensionality: cross-nosological and multimodal cardiac interoceptive deficits.	Abrevaya et al. (34)	Examine the impact of neural relative to autonomic disturbances of cardiac interoception across neurological conditions.	Data from 149 participants divided between two pathological groups (neurological and cardiac) and controls.	Data-driven approach to evaluate the relevance of the cardiac interoceptive dimensions in the discrimination of neurological and cardiac pathologies. A classification pipeline was used with the input from behavioral dimension and different levels of analysis.	This study demonstrates the possibility of computational models to integrate different systems (cardiac and neurologic) to find relevant variables for the discrimination of disorders. This study reached to mentioned conclusions by implementing different automatized analyses including support vector machines and machine learning procedures.
Thought disorder measured as random speech structure classifies negative symptoms and schizophrenia diagnosis 6 months in advance.	Mota et al. (54)	Determine if early markers of speech disorganization during recent-onset psychosis measured using SpeechGraph could correctly classify the severity of negative symptoms as well as the schizophrenia diagnosis.	Graph measures of different memory reports.	Data-driven software to measure graph attributes of connected speech.	This study has a different approach to the use and application of computational models. It takes a software made through a data-driven approach to arrive at quantitative measurements of formal thought disorder. This could in turn help to delimitate better these alterations to make a more precise diagnosis. There are other applications of this software (53, 55, 56, 110, 155, 156).
A computational framework for the prioritization of disease-gene candidates.	Browne et al. (72)	Evaluate the performance of a method for gene prioritization applied to Alzheimer’s disease.	Gene Expression Omnibus (GEO) database.	Model-based approach based on network theory for the creation of Protein–Protein Interaction Networks (PPIN). Integration of multiple datasets for the construction of PPIN.	A framework that integrates diverse heterogeneous data including gene expression and network topological features to prioritize and analyze disease-gene candidates applied to AD as a Case Study. Demonstration that the integration of PPINs along with disease datasets and contextual information is an important tool in unraveling the molecular basis of diseases.
Integrated co-expression network analysis uncovers novel tissue-specific genes in major depressive disorder and bipolar disorder.	Han et al. (73)	Explore the expression specific characteristics of different areas by systematic analysis of larger samples of brain tissues and determine gene expression patterns and tissue-specific expression profiles between major depressive disorder and bipolar disorder.	Transcriptomic datasets retrieved from the Gene Expression Omnibus (GEO).	Data-guided approach with a weighted gene co-expression network analysis to construct gene co-expression networks for large scale gene expression profiling from various regions of the brain.	Give insights in the tissue-specific functions of various brain regions in the context of psychiatric disorders (MDD and BD). It is a report on functional similarities and specificities between tissues of two psychiatric disorders.
Dissecting psychiatric spectrum disorders by generative embedding.	Brodersen et al. (102)	Examine the feasibility of defining subgroups in psychiatric spectrum disorders by generative embedding.	Functional MRI dataset performing a working memory task.	Theory-driven approach through the use of generative embedding. The researchers used parameter estimates from a dynamic causal model (DCM) of a visual-parietal-prefrontal network to define a model-based feature space for the subsequent application of supervised and unsupervised learning techniques.	This is a proof-of-concept study to examine how model-based clustering could be used to dissect psychiatric spectrum diseases into physiologically defined subgroups, giving foundation to possible implications in the delivery of precision psychiatry. It gives insight into the constraints of a model-guided approach according to its assumptions.
Uncovering social-contextual and individual mental health factors associated with violence via computational inference.	Santamaría-García et al. (103)	Evaluate individual mental health and sociocontextual determinant of violence simultaneously and explore their association to different domains of violence.	Data was taken from a sample of 26,349 ex-members of Colombian illegal armed groups who entered programs of transitional justice for reincorporation into civilian life. They responded to a semi-structured interview designed by the Agency for Reintegration and Normalization.	Combination of theory- and data-driven approaches of examination and analysis of historical records of ex-members of illegal armed groups in Colombia, using deep learning and machine learning methods to identify the most relevant factors associated with domains of violence.	This study investigates the interaction of contextual and individual factors associated with violence in the Colombian context with novel methodologies to take into account historical assessments. Another important aspect of this study is the usage of a combination of theory- and data-driven approaches. This study is not focused in a mental disorder, however it has been weighed the importance of social and individual mental health variables like violence.
Predicting suicide attempts and suicide deaths following outpatient visits using electronic health records.	Simon et al. (157)	Develop and validate models using electronic health records to predict suicide attempt and suicide death following an outpatient visit.	Health care records from seven health systems of 2,960,929 patients.	Data-driven approach to develop prediction models, which were separated between mental health specialty and primary care visits.	This study describes an analysis of a great amount of data across different health care systems. Within the supplementary material, there is a public repository including specifications and code for defining predictor and outcome variables alongside a data dictionary and descriptive statistics for analytic data sets, which impact the reproducibility of the study.
Speech structure links the neural and socio-behavioural correlates of psychotic disorders.	Palaniyappan et al. (53)	Investigate the neural basis and the functional relevance of the structural connectedness of speech samples of subjects with schizophrenia and bipolar disorder.	Clinical assessments of 34 patients with schizophrenia and 22 with bipolar disorder.	Data-driven software to measure graph attributes of connected speech.	This study exemplifies the possibility of establishing a relationship between pathological phenomenology and biological markers. This opens up the possibility of integrating this tool with other computational approaches to achieve a multilevel analysis.
Pattern of neural responses to verbal fluency shows diagnostic specificity for schizophrenia and bipolar disorder.	Costafreda et al. (109)	Through the usage of the verbal fluency task, the researchers investigated the functional neuroanatomy of executive function in schizophrenia and bipolar disorder. The hypothesis was that the pattern of regional brain responses would correctly identify the diagnosis for each participant at the individual level.	Patients with schizophrenia and bipolar disorder in remission. They were subjected to a clinical assessment and were taken fMRI.	Data-guided approach with the use of machine learning to conduct a pattern classification analysis.	The study highlights the possibility of being able to integrate data from a neurocognitive task and reveal its neurobiological basis to determine precisely diagnostic differences between different clinical entities. It also highlights that the difference between diagnosis comes from degrees of functionality and the limitation of discriminating between them.
Prediction models of functional outcomes for individuals in the clinical high-risk state for psychosis or with recent-onset depression: a multimodal, multisite machine learning analysis.	Koutsouleris et al. (112)	Determine whether predictors associated with social and role functioning can be identified in patients in clinical high-risk states (CHR) for psychosis or with recent-onset depression (ROD) using clinical and imaging-based determinant with machine learning analysis. Assess the geographic, transdiagnostic and prognostic generalizability of machine learning and compare it with human prognostication. Explore sequential prognosis encompassing clinical and combined machine learning.	116 patients in CHR states and 120 patients with ROD.	Data-driven approach using machine learning. Three models of prediction were used (one with clinical variables, one with neuroimaging variables and one integrating the other two).	This study not only explore the predictive model from a data driven approach, but it was also geographically validated. The researchers tested the transferability of the model to other outcomes. It also takes into account the reliability of the inputs which were feeding the model. This study inquires about social factors that drive the personal and socioeconomic burden of psychotic and mood disorders integrating clinical and brain structural data.
Natural language processing of clinical mental health notes may add predictive value to existing suicide risk models.	Levis et al. (113)	Determine if the use of natural language processing (NLP) in psychotherapy note text can provide additional accuracy over currently used suicide prediction models (REACH VET).	Data from the Department of Veterans Affairs (VA) of patients newly diagnosed with PTSD between 2004 and 2013.	Data-driven approach which uses NLP to evaluate unstructured electronic medical records of a sample from de VHA PTSD treatment population.	The method presented in this paper introduces to a dynamic model that helps identify and monitor predictor variables and how they change over time. This gets closer to an ecologically valid tool to asses an individual. This type of approaches on NLP have been used in other pathologies like delirium (158), Alzheimer’s disease (159, 160), schizophrenia and others (161, 162).
A machine learning approach using EEG data to predict response to SSRI treatment for major depressive disorder.	Khodayari-Rostamabad et al. (163)	Evaluate the performance of a machine learning methodology based on the pre-treatment electroencephalogram for prediction of response to treatment with SSRI in patients with MDD.	Subjects with MDD derived from a tertiary Mood Disorders Clinic. They were all considered treatment resistant.	Data-driven approach using machine learning to select the most discriminating features from EEG. Then, these features are fed into a classifier based on a mixture factor analysis to give a likelihood value.	This study exemplifies a possible approach to improve treatment in a personalized manner in line with precision psychiatry.
Cross-trial prediction of treatment outcome in depression: a machine learning approach.	Chekroud et al. (119)	Develop an algorithm to assess whether patients will achieve symptomatic remission from a 12-week course of citalopram.	Data was collected from a STAR-D sample.	Data-driven approach using machine learning to identify which variables were most predictive of treatment outcome.	This study determines the possibility of using computational approaches to mine existing clinical trial data to improve on accuracy of risk or treatment response prediction. However, this model only predicts response to specific drugs. There has to be a contextualization of the applicability of the model.
Gyri-precise head model of transcranial direct current stimulation: improved spatial focality using a ring electrode versus conventional rectangular pad.	Datta et al. (123)	Compare the focality of conventional rectangular-pad stimulation with ring electrode configuration using a MRI-derived head model.	Models of two electrode configurations.	Use of a head model to predict relative spatial focality and the influence of tissue geometry/conductivity.	This study demonstrates a way of translate computational models of variables associated with treatments such as direct current stimulation to clinical applications through the design and optimization of treatment variables.
Effects of modifying the electrode placement and pulse width on cognitive side effects with unilateral ECT: a pilot randomized controlled study with computational modelling.	Martin et al. (124)	Determine if the frontoparietal placement of electrodes improves retrograde memory outcomes compared to temporoparietal placement.	Patients recruited from a single hospital in Sydney.	Computational model (164) was used in a subset of participants to determine if higher levels of stimulation in regions of interest would be related to worse or better cognitive outcomes.	This study gives an example of how data from computational models could be integrated to results from clinical investigations to individualize treatment options such as DCS.
Patient-specific analysis of the volume of tissue activated during deep brain stimulation.	Butson et al. (125)	Develop and test a methodology that would enable prediction and visualization of the volume of axonal tissue activated during DBS.	One patient with Parkinson’s disease.	Patient-specific model of STN DBS for PD and the VTAs. This model was constructed from 3D brain atlas that was warped to the patient MRI using a non-linear warping algorithm. The electrical and biophysical models rely on finite element models.	This model integrates anatomical, electrical, and biophysical representation of DBS. It also integrates simulation data with clinical data from subject. The limitation of this model is the evaluation of only one patient.
Functional connectome fingerprinting: identifying individuals using patterns of brain connectivity.	Finn et al. (126)	Determine if functional connectivity profiles can act as an identifying fingerprint capable of identifying an individual from a set of connectivity profiles.	Data collected from the Human Connectome Project.	Data-driven approach using a group-wise spectral clustering algorithm for the definition of networks capable of being compared to each other. This correlation was made through the use of whole-brain connectivity matrix.	This study gives the foundation for novel test inferences about functional brain organization can relate to distinct behavioral phenotypes. The discriminating power evidenced in this study is partly the result of the relatively long period of time of follow-up. This can be integrated in frameworks like RDoC. It also gives the base for neuroimaging studies which rely on single subjects, beyond population-level studies.
Linked dimensions of psychopathology and connectivity in functional brain networks.	Xia et al. (127)	Identify brain-based dimensions of psychopathology.	Datasets taken from the Philadelphia Neurodevelopmental Cohort (PNC).	Data-driven approach based on sparse canonical correlation analysis.	This study uses network theory to construct patterns of functional connectivity, which could be linked to transdiagnostic dimensions of psychopathology. In this study, these patterns displayed developmental and sex differences. This in turn tackles the problems of comorbidity and heterogeneity previously discussed in this article.
Origin of cyclicity in bipolar disorders: a computational approach.	Goldbeter (131)	Evaluate a model for bipolar disorders based on mutual inhibition of two putative neural circuits governing the affective syndromes.	Mathematical model based on reciprocal inhibition.	Theory-driven approach of a mathematical model to predict the cyclicity of bipolar disorders. This model is based on a phenomenological model.	This article gives an example of translating a phenomenological level to mathematical terms in order to explain and predict a characteristic of a phenomenon (cyclicity of bipolar disorders).
Computational mechanism of effort and reward decisions in patients with depression and their association with relapse after antidepressant discontinuation.	Berwian et al. (132)	Establish whether the decision to invest effort for rewards represents a persistent depression process after remission.	Sample of patients in a Swiss and German university setting.	Theory-driven approach where a generative computational model was used to represent the putative computations of the behavioral pattern.	This study explores a computational model for effortful behavior applied in a sample of patients with depression. This gives a straightforward manner to assess this behavioral feature and find associations that are important form a prognosis and treatment perspective. Nonetheless, this study has limitations from a replicability perspective.
Making working memory work: a computational model of learning in the prefrontal cortex and basal ganglia.	O’reilly and Frank (133)	Presentation of a computational model of working memory based on the prefrontal cortex and basal ganglia.	The 1-2-AX task.	Theory-driven approach which uses a reinforcement learning mechanism.	This paper describes how a theory-driven model is constructed from data previously acquired which is integrated to elucidate a specific process.
Towards a computational psychiatry of juvenile obsessive-compulsive disorder.	Loosen and Hauser (134)	Review computational, neuropsychological and neural alterations in juvenile OCD. Link these findings to adult OCD. Establish a neurocomputational framework that illustrates the development of symptoms in the context of juvenile OCD.	Narrative review.	Theory-driven approach based on a narrative review of computational, neuropsychological and neural alterations in juvenile OCD. The framework proposed is based on a meta-controller with different rates of maturation of complex systems.	This study describes a proposition of a theory-driven model for the development of obsessive symptoms. However, this model is only speculative and requires further investigation to be validated. It highlights the importance of the *a priori* knowledge to construct the model and the dependance on inputs to determine the strength of the model.
Adaptive current-flow models of ECT: explaining individual static impedance, dynamic impedance, and brain current density.	Unal et al. (138)	Examine the relations between the physical properties of the ECT stimulus, patient head anatomy, and patient-specific impedance to the passage of current.	Clinical data from a trial series.	Theory-driven approach. The researchers develop an individualize (MRI-derived) finite element method (FEM) to model transcranial electrical stimulation with dynamical changes in tissue conductivity.	This model gives the opportunity of studying parameters that have been proposed as important factors in the therapeutic response (165), but they are difficult to study under a “normal” clinical study.

Finally, it is essential to highlight that these applications go beyond the nosology provided by the DSM and allows the visualization of phenomena that can impact the course and prognosis of these disorders or the mental health of individuals in general. An example of this is creativity, which can be understood as the ability to create unique products such as artists (Creativity with a capital C); or as a cognitive function that helps the individual adapt to his environment and give answers to his environment (creativity with c) (140). The latter, in turn, depends on divergent and convergent thinking (141). In the review carried out by Mekern et al. (142), it can be evidenced how the same phenomenon can be studied from different levels and segmented into other processes even going so far as to predict or determine how these processes would be affected by specific alterations or disruptions. With the help of computational modeling, it improves its understanding.

## RDoC: Possible response to the constraints of nosological systems

The nosological systems encountered in the clinical and research practice delineate highly heterogeneous phenotypes that lack reliability and validity, which has restrained advancements in the field as the computational tools rely on the input one puts in them (82, 143). In this way, if one takes invalid or erroneous input to a model, which can be valid, the data that results from this process is also invalid and could deviate the researcher to a categorical error. The necessity for a system of categorizing these problems and disorders in a way that conceptualizes them as a mixture of interacting and dimensionally varying processes is at the front and center of the problem (87). This, in turn, could give us a way of representing these problems in a more ecologically valid way.

Different approaches have been made by researchers in order to arrive at solutions to these limitations. One of them is RDoC. The *Research Domain Criteria Project* was initiated by the NIH (National Institute of Health) to address the different problems that research has encountered in mental health, specifically mental health disorders (144–146). This project was conceptualized as a research framework, so it has no applications in clinical nosology, nor does it pretend to be a replacement for it. Although, one of the potential impacts is to achieve a classification system with a more significant neurobiological basis without leaving a biological reductionist vision of these disorders. It recognizes that mental disorders are multicausal, mediated by biology (brain). In addition, the RDoC is structured as a matrix with different units of analysis, which are grouped into research domains. These domains are viewed longitudinally, influenced by neurodevelopment and the context in which they are imbued. Computational psychiatry, then, introduces itself as a great tool in this type of initiative, aligning with its principles, since it allows to appreciate of shared mechanisms between cognitive alterations, psychopathological domains, and disorders (59), achieving integration between the different levels of analysis (units of analysis and domains). It does this by finding objective, observable, and measurable characteristics organized into taxonomies outside current nosology (25), achieving a more solid basis for neurobiological research.

With this initiative, it has been possible to see that in most mental disorders, there is an overlap between neural circuits in which the processing of threats (amygdala, hippocampus, orbitofrontal cortex, and ventromedial prefrontal cortex), rewards (amygdala, ventral tegmental area, locus coeruleus, and nucleus accumbens) and perception of stimuli (thalamus, sensory cortex, and inferior frontal gyrus) are counted (25). This suggests that mental disorders may be due to different modes of dysregulation of control processes. That is a different dynamic system. These altered processes can occur from the cellular and molecular level to the level of circuits. And this generates a greater difficulty since the alterations will only vary qualitatively but quantitatively. This, at a practical level, limits the possibility of using only clinical judgment to determine these nuances. Again, the problem with these ambiguous cases, which are the rule and not the exception in psychiatry, is highlighted by the lack of persistence in diagnosis given to a person over time and the problem of comorbidity and heterogeneity (147).

However, it does present guidelines that can be a response to the criticism previously mentioned of nosology and psychiatric research based on it, as well as a bridge for using computational models to the approach of multidimensional and hierarchical organization of mental functions, the non-linear dynamic interaction between the components of the system and its heterogeneity. Thus, computational psychiatry aligns with one of the objectives of the RDoC, which is to improve the accuracy of the phenotypes and their alignment with highly plausible biological and cognitive models based on experimental settings used in neuroscience research applied to psychiatry (87).

Nonetheless, this is one of many models which have risen to deal with the limitations and constraints previously described. The HiTOP is a data-driven, hierarchically based organization of psychopathology (86). It conceptualizes psychopathology as a set of dimensions organized into increasingly broad, transdiagnostic spectra. This is made by using factor analysis between different symptoms to generate a taxonomy of mental disorders. In these scenarios, the computational models would aid in determining these psychopathologic patterns using path analyses in clinical datasets. Lastly, they would also be helpful in establishing psychopathologic patterns taking into account their context (social influences and contextual factors).

Another alternative is the network theory based on pattern analysis, similar to computational psychiatry. To construct these networks, one has to analyze a significant amount of data that can capture cohesion, coherence, and patterns of synchrony (148). In this sense, computational psychiatry dialogues with the network approach both require massive data processing to formulate theoric models.

As previously discussed, HiTOP is another proposed model for this endeavor. It was constructed through factor analysis and latent class analysis to organize psychopathology according to the natural covariance structure between symptoms, maladaptive behaviors, and traits (61, 86). This model focuses on the psychopathological level remaining agnostic to the underlying phenotypes encountered. Moreover, it can be a tool to aid RDoC-informed research by providing psychometrically valid data to reach more robust psychiatric phenotypes (149), and in doing so, it can ameliorate the computational models used in clinical and research fields.

## General limitations

One of the limitations that must be considered in the explanatory models is that the data previously collected empirically may contain significant biases that prevent distinguishing between different hypotheses of the mechanisms that generate psychiatric dysfunctions. For this reason, it is of the utmost importance to recognize parameters that allow discriminating between models (59). On the other hand, for data-driven approaches, the clinical datasets from which one can take the information are limited in data quality, organization and accessibility, making it difficult to get the data for the machine learning algorithms (100, 150).

Another limitation is inherent to mental disorders since they usually present dysfunctions or deficits that are generalized, shared by many disorders, and only differentiated at a quantitative level (59), so a large enough sample must identify these differences. This limitation can be overcome by the formation of consortiums like the ones developed for genomics studies and others (151).

Still, another limitation is that, in most cases of mental disorders, the brain regions or alterations underlying a particular dysfunction have not been accurately determined. However, reverse-engineering strategies can overcome this automatically, seeking to identify physical and biological laws through data (25). However, this raises another problem because these structures can be purely mathematical entities that do not have a basis in biological structures.

In addition to this, neurobiological models describe data as unreliable, meaning that the probability of error in the model must be quantified (25). These errors are critical in these models, where many interrelated variables spread that error to different parts of the system. Typically, this type of error is controlled by increasing the sample size; however, in this case, it would worsen the problem because it could result in inaccurate models with statistical significance (25). Moreover, brain processing is non-linear, having complex interrelationships, amplifying, or decreasing the noise of the inputs. This causes linear regressions to lose their significance. In neurobiological responses, various processes like serial signaling processes, thresholds, filters, saturation, feedback, etc. All of these are non-linear and, therefore, more difficult to describe.

Also, using Bayes’ theorem to choose the best model will often lead to models that do not describe the best generative model. Therefore, one should always validate the model (35) and always keep in mind the possibility of finding better models.

## Conclusion

Computational psychiatry can be a tool for understanding mental health. This involves a great effort, which requires the articulation of multiple disciplines and different levels of analysis. Therefore computational psychiatry could become a high point and central to attempts such as RDoC or ROAMER (Roadmap for Mental Health Research in Europe) (152) to achieve a better conception of both mental disorders and mental health, with the articulation with other models like HiTOP.

To achieve this, it is necessary to overcome previously evidenced obstacles such as heterogeneity and comorbidity, together with the acceptance and use of the complexity of these systems with non-linear dynamics, making use of tools that allow us to understand it in a way in both biological and psychological reductionist perspectives are not given. In addition, the opportunity opens up to begin the study, articulation, and integration with factors that modulate the presentation and prognosis of mental disorders but that are left to the context and have been covered only tangentially, as are social processes such as violence, abuse or forced displacement. The development of research capacity achieves a better assessment of the needs for the care of the population, increasing knowledge about the effectiveness of different interventions and creating a critical mass that is essential for the development of the scientific debate on various topics in mental health (153). In addition, this theoretical framework model how the subject acquires and transforms their internal cognitive processes to give rise to their behavioral responses, which are observed in clinical practice (15).

However, to accomplish all these promises, several limitations must be considered. The necessity for not only replicating the results of different investigations arises with the need for reproducible investigations to tackle the falsifiability problem. In this same direction, with the growth of analytic power, the possibility of finding associations that are not significant or valid also increases. So, the validation of these models is yet another fundamental aspect that must be tackled by researchers.

Finally, computational psychiatry would allow us to provide better care for mental health problems in primary care. This considers that the burden of patients increases with poor support for the number of professionals in mental health. Then, the models given by computational psychiatry would allow the specialist to have better visualization and contextualization of each patient’s specific case considering multiple factors that often cannot be given enough weight due to restrictions. Also, data-based computational models allow predictions or diagnostics, and these responses can be better adjusted to the context of each country and can be free or require low investments.

**FIGURE 1:**
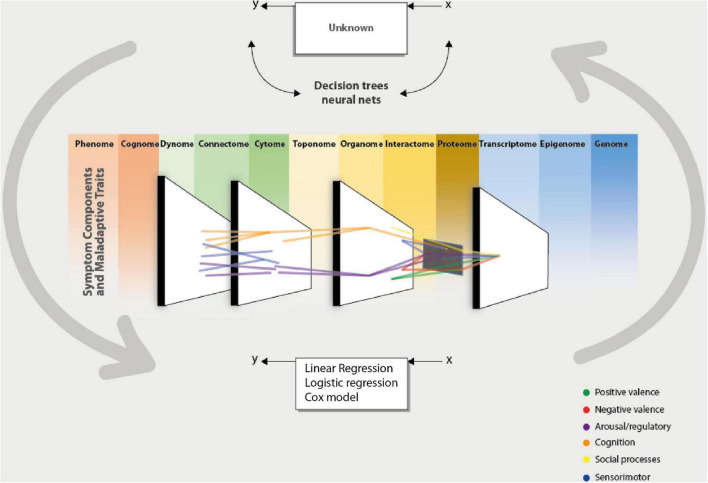
Computational psychiatry aids the clinician and the researcher in integrating data from different sources of information, which could be taken from the omics perspective. This integration is made possible by complementing the data modeling culture using the algorithmic modeling culture proposed by Breiman (43). This permits the validation of models or data that can be measured by predictive accuracy. By taking these inputs and processing them through a computational system (algorithm), one could present data-driven or theory-driven responses to clinical and research questions. This enables us to bring forward an integrative and cohesive framework associated with others. The network theory can integrate the different units of analysis (scale level) of a phenomenon or give a cohesive picture of the interaction between different domains. And in turn, this could give us a more precise phenotype to arrive at a dimensional conception (HiTOP). These computational approaches to understanding psychiatry represent the brain’s functioning [phantastic organ (20)]. In other words, using computational approaches to comprehend psychiatry mimics the normal functioning of the statistical machine we call the brain.

The promises are manifold, but their success depends on their applicability and the possibility of generating translational knowledge (154), Figure 1 proposes a framework to arrive at this result.

## Author contributions

JC conceived the general idea of the review and summarized the data found through the search, which was edited by HS-G. Both authors developed the search criteria to complete the review, analyzed the data found, and revised it critically to arrive at the approved version to be published.
